# Revision of 116 orthognathic surgery patients operated on with the high-oblique sagittal osteotomy (HOSO): a retrospective case series (PROCESS-compliant article)

**DOI:** 10.1007/s00784-020-03653-2

**Published:** 2020-10-26

**Authors:** C. Herrera-Vizcaino, L. Seifert, M. Berdan, S. Ghanaati, M. Klos, C. Landes, Robert Sader

**Affiliations:** 1 Clinic for Maxillofacial and Plastic Surgery, Johann Wolfgang Goethe University, Frankfurt Am Main, Germany; 2 Department of Oral, Maxillofacial and Facial Plastic Surgery, Sana Klinikum, Offenbach am Main, Germany

**Keywords:** Orthognathic surgery, High oblique sagittal osteotomy, HOSO

## Abstract

**Background:**

The high-oblique sagittal osteotomy (HOSO) is an alternative to a bilateral sagittal split osteotomy (BSSO). Due to its novelty, there are no long-term studies which have focused on describing the incidence and type of complications encountered in the post-operative follow-up. The aim of this retrospective study is to analyze patients operated on with this surgical technique and the post-operative complications encountered.

**Patient and methods:**

The electronic medical records of all patients treated with orthognathic surgery at the Department of Oral, Maxillofacial and Facial Plastic Surgery, University Hospital Frankfurt, Goethe University, Frankfurt, Germany, between the years 2009 and 2016 were retrospectively reviewed.

**Results:**

A total of 116 patients fulfilled the inclusion criteria. The cases operated on with the standard osteosynthesis (X, Y, and straight) showed a complication rate of 36.37% (*n* = 4/11). The cases operated on with the HOSO-dedicated plates (HOSO-DP) showed, in total, a complication rate of 6.67% (*n* = 7/105). The most common post-operative complication resulting from both fixation methods was a reduction in mouth opening and TMJ pain for 4.3%. During the first years of performing the surgery (2009–211), a variety of standard plates had material failure causing non-union or pseudarthrosis. No cases of material failure were observed in the cases operated on with the HOSO-DP. The statistical results showed a highly significant dependence of a reduction in OP-time over the years, when the HOSO was performed without additional procedures (*R*^2^ > 0.83, *P* < 0.0015).

**Conclusion:**

The rate of complications in the HOSO were shown to be comparable to the rate of complications from the BSSO reported in the literature. Moreover, the use of the ramus dedicated plate appears to provide enough stability to the bone segments, making the surgery safer.

**Clinical relevance:**

The HOSO needs to be considered by surgeons as an alternative to BSSO. Once the use of the HOSO-DP was established, the rate of complications and the operation time reduced considerably.

## Introduction

Orthognathic surgery is a surgical technique used for the correction of malocclusions caused by mandibular prognathism, retrognathia, open bite, and asymmetries. The most common surgical design used is the bilateral sagittal split osteotomy (BSSO). This technique was described by Obwegeser and modified by Dal Pont in 1961 [1, 2]. An alternative to BSSO is the high-oblique sagittal osteotomy (HOSO), which was initially described as the Schlossman osteotomy [3]. During its introduction in 1922, this technique encountered complications mainly related to the lack of adequate osteosynthesis material and the surgical management of the remaining proximal segment. Additionally, it was traditionally carried out traumatically with a Gigli or Feldmann saw. However, due to the development of new osteosynthesis materials and devices like the piezotome, this osteotomy design is gaining higher acceptance among surgeons. The design of the osteotomy and the fixation methods have gone through several modifications. Most recently, Landes et al. described the HOSO with an intraoral approach and a 45°-angled osteotomy of the mandibular ascending ramus using a piezotome. The piezoosteotomy of the mandibular ramus begins laterally at the occlusal level and ends medial above the alveolar foramen. This provides larger bone segments with better adapted surfaces, reduces recurrences, makes reossification safer, and enables a greater rotation of the occlusal plane than the traditional HOSO technique. The author named the procedure a low-to-high oblique piezoosteotomy, and it remains the latest modification described in the literature [4]. Furthermore, studies using computer models have provided insights regarding the stability of the bone segments and the osteosynthesis material. In a recent study using a computer biomodel and finite element analyses, three sets of commercially available osteosynthesis material were evaluated in order to ascertain which provided the best stability to the bone segments. The sets used were three screws horizontally placed, 2 straight plates 2.0, and a ramus dedicated plate. Although all sets of osteosynthesis were enough to support the bone segments, the 3-screw set showed a higher stress concentration over the screws, bone, and coronoid process. The main described advantages of the HOSO technique are namely the simplicity of the surgery that makes it easy to learn; the reduced risk of neurosensory alterations, because of a higher protection of the inferior alveolar nerve, compared with the BSSO; and the fact that it does not compromise the function of the temporomandibular joint [5]. Many studies have described the incidence of complications related to the BSSO [6, 7]. Such complications include temporomandibular joint pain, infection, alveolar nerve disturbances, fixation material loosening, periodontal disease, and pseudoartrosis [6]. In the case of the HOSO, the main concerns continue to be condylar positioning and temporomandibular joint (TMJ) function, bone contact area, and neurosensory alterations. However, regarding the TMJ function, positioning control of the proximal segment was performed on 22 patients during and after surgery. The authors of the study reported no alterations in the condyle positions [8]. These results were further confirmed in an additional study by evaluating the post-operative computed topographies of 40 patients. It was shown that there were no alterations in the condylar position when comparing the BSSO and the HOSO, so the selection of one technique over the other should not be based on this parameter [9].

Due to the novelty of the HOSO, there are no long-term studies which focus on describing the incidence and type of complications encountered post-operatively. The aim of this retrospective study is to analyze the patients who received interventions using the latest modification of this surgical technique and to identify the post-operative complications encountered.

## Patients and methods

The study is a single centered, retrospective, and consecutive case series. The study was conducted following the process guidelines [10]. The electronic medical records of all patients treated with orthognathic surgery at the Department of Oral, Maxillofacial and Facial Plastic Surgery at the University Hospital Goethe of Frankfurt, Germany, between the years 2009 and 2016 were retrospectively reviewed. The identity of the patients was kept from the authors during the extraction of the data. Patients fulfilled the inclusion criteria if they underwent orthognathic surgery to correct malocclusion using the HOSO. Additionally, radiographic assessment before and after surgery either by ortopantomography (OPG), computer tomography (CT), or magnetic resonance imaging had to be available. The letter was also used to analyze the reasons for postoperative TMJ problems resulting, i.e., from a dislocated proximal segment or disk. Moreover, post-operative sensory dysfunction was recorded during the follow-up performed at 3 month post-operatively. Patients were questioned about neurosensory alteration while sitting with their eyes closed. Light touch sensation and pin prick sensation were examined using 4.0 suture material and a dental probe. The responses were recorded as absent or positive subjective perception. Static 2-point discrimination was tested using a blunt end of an orthodontic gauge and recorded as correct, incorrect, or no perception. Patients with a history of systematic disorders (muscular dystrophy, myasthenia gravis, osteogenesis imperfect, and previous reports of TMJ pathologies) and with previous mandibular trauma were excluded. Extracted records included age, gender, year of operation, operation time (OP) of a mono-und bimaxillary surgery, OP-time of patients operated on with a HOSO without any other procedure, the type of osteosynthesis material used dived in standard plate (SP; representing X, Y, and straight plate), and HOSO-dedicated plate (HOSO-DP) and post-OP complications during the follow-up. All procedures were performed by senior maxillofacial surgeons (SR and LC). Osteotomies in the mandibula were performed using piezosurgery® with sonographic control to correctly position the condyle intraoperatively. Particular attention was paid to the stability over time of the osteosynthesis material in regard to fracture of the osteosynthesis.

**Fig. 1:**
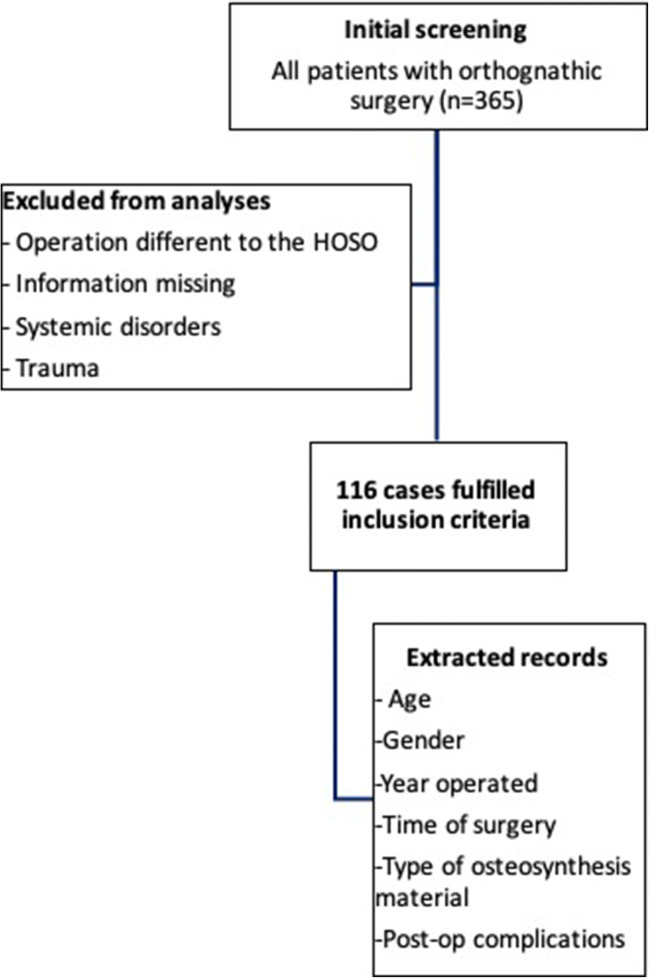
Flowchart of case-selection criteria

A simple linear regression was calculated to predict the correlation between a HOSO with or without additional procedures with respect to a reduction in the OP-time (dependent variable) over the years. A student *t* test was used as the statistical method for the determination coefficient (*R*^2^) at a 95% confidence interval. Data was checked for normality of residual with a D’Agostino-Pearson omnibus (K2) test. The primary endpoint was the coefficient of determination, which was considered statistically significant if *p* < 0.05. Data analyses were carried out using GraphPad Prism 8.0 software (GraphPad Software Inc., La Jolla, CA, USA). The results are presented as the mean and standard deviation (SD) and depicted in graphs. A flow chart describing the study methodology was depicted following the PROCESS guidelines (Fig. 1).

## Results

**Fig. 2:**
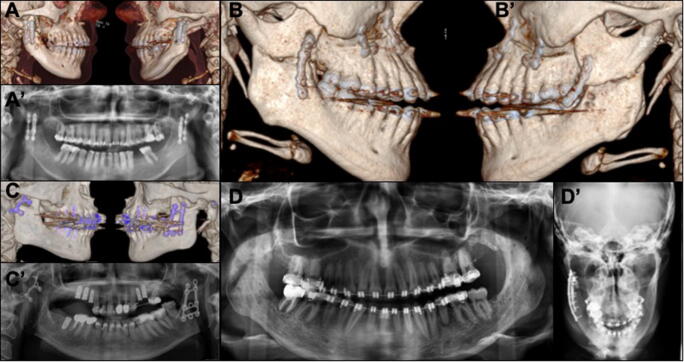
2D images (orthopantomography) and 3D reconstructions (computer tomography) of patients operated on with the high-oblique split osteotomy (HOSO) complicated with failure of the osteosynthesis material. 3D reconstructions show a right and a left view of the patients. (A-A′) Displacement of the proximal segment. (B-B′) Loosening of the osteosynthesis material. (C-C′) Displacement of the proximal segment on the right side. (D) Ramus fracture and pseudarthrosis after the removal of the osteosynthesis material. (D′) Management of the complication by providing new osteosynthesis material

**Fig. 3:**
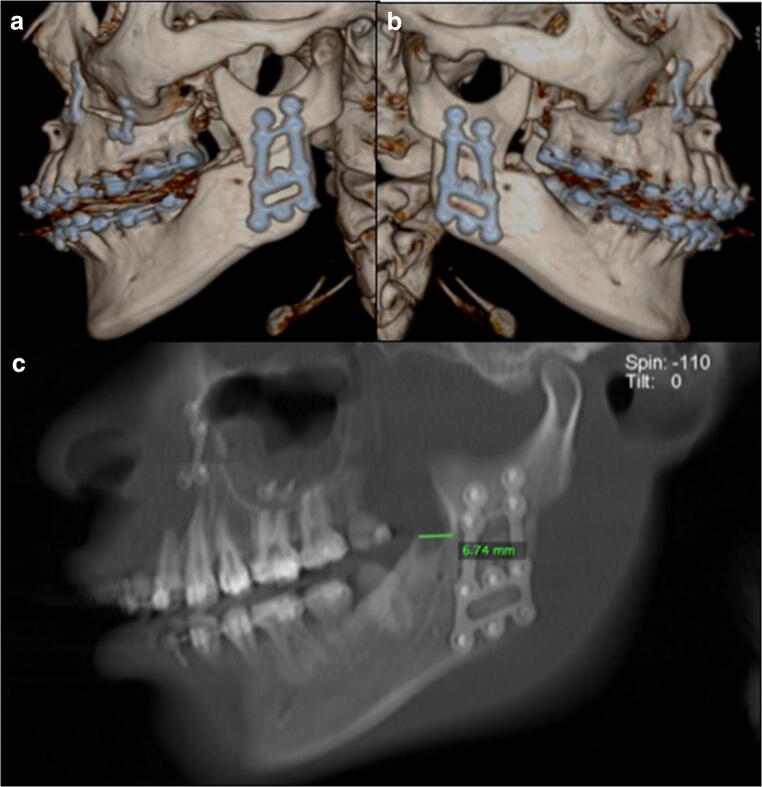
Ideal conditions of a patient operated on with the high-oblique split osteotomy (HOSO). An advancement of 6.74 mm was performed without reported complications during the post-operative follow-up. **a** Left view. **b** Right view. **c** Sagittal cross-section

**Fig. 4:**
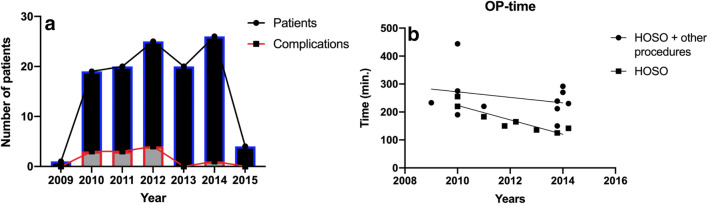
Complications related to the HOSO. **a** The number of patients and complications per year. **b** Simple linear regression showed highly significant dependence of the reduction in OP-time over the years, when the HOSO was performed without additional procedures (*R*^2^ > 0.83, *P* < 0.0015)

The surgeries were performed between October 2009 and April 2016. From a total of 365 patients, 116 patients fulfilled the inclusion criteria. The majority of patients were in their 20s (52.5%) (Table 1). There was an increase in the number of patients operated on during the year with the highest number in 2014. The number of patients was evenly distributed between genders (Table 2). In total, patients were checked up on with follow-ups over a span of 1 year, weekly in the first months, then after 3 months and finally after a year. Seventy-two patients presented a class III malocclusion and were treated with mandibular set-back, while 42 patients presented a class II malocclusion and were treated with mandibular advancement surgery. Information regarding millimeters of advancement, setback, or rotational movements were not found in the records. Twenty-two patients underwent mandibular osteotomy (monomaxillary surgery) together with additional procedures, i.e., implant insertion, frenectomy, or third molar extraction. The overall mean OP-time was 213.5 ± 67.4 min. From these 22 patients, 8 received mandibular osteotomy without any other surgical procedures and the mean OP-time was 157.4 ± 45.1 min. The remaining patients (94) underwent a bimaxillary procedure, and the OP-time was 315 ± 112.4 min. During the first 3 years, between 2009 and 2011, the cases where the SPs were implemented showed a complication rate of 36.37% (*n* = 4/11). The cases operated on with the HOSO-DP showed, in total, a complication rate of 6.67% (*n* = 7/105) (Table 3). Cases of plate failure and non-union or pseudarthrosis were treated with open reduction, debridement (removal of fibrous tissue), and grafted with iliac crest or mandibula ramus graft. The internal fixation was replaced with the HOSO-DP or reconstruction plates when necessary (Fig. 2). The accumulated complication rate reduced in 2013 and 2014 to 2%. No neurosensory alteration or nerve injuries were recorded in the 116 cases analyzed. The most common complication during the follow-up was TMJ pain with 5 cases (Table 3). Four of those patients were operated on with the HOSO-DP and presented pain (*n* = 1) or reduction in mouth opening (*n* = 3) after 6–8-month post-OP. They were treated with arthrocentesis of the TMJ as well as physical therapy which led to an improvement of the clinical symptoms. One patient operated on with an SP presented a reduction in mouth opening and was also treated with arthrocentesis of the TMJ and physical therapy. One case of mandibular fracture occurred after the removal of the HOSO-DP 8 month post-operatively. Additionally, plate removal was necessary because of an event of infection 6-month post-OP. The infected area was surgically treated, and the plate was removed. The patient received i.v. antibiotics over 5 days and discontinued on dismissal. In total, 11/116 cases of complications were found which accounted for 9.4% of all patients operated on. During the first years of performing the surgery (2009–2012), a variety of fixation plates with different designs were used (Fig. 2a–d). However, the most common plate used was the HOSO-DP (Modus Orthognatic Medartis®, Basel, Switzerland) (Fig. 3). Figure 4b corresponds to the statistical results showing a highly significant dependence of the reduction in OP-time over the years, when the HOSO was performed without additional procedures (*R*^2^ > 0.83, *P* < 0.0015) (Fig. 4).

**Table 1:** Age distribution of patients operated on with the HOSO allocated by decades

Age range (year)	Patients (n)	Ratio (%)	Complications (*n*)
14–19	25	21.5	2
20–29	61	52.5	6
30–39	17	14.6	1
≥ 40	13	11.2	2
Total	116	100	11

**Table 2:** Gender distribution

Year	Male (*n*)	Female (*n*)	Complications (*n*/%)
2009–2010	12	8	2 (10.0%)
2011–2012	19	26	8 (17.8%)
2013–2014	20	27	1 (2.1%)
2015	2	2	0 (0.0%)
Total	53	63	11/116 (9.4%)

**Table 3:** Number of complications from the total number of patients associated with the osteosynthesis material. Standard plate (SP; representing X, Y and straight plate) and high oblique sagittal osteotomy dedicated plate (HOSO-DP) (Medartis MODUS Orthognathics®, 2.0 mm, Basel, Switzerland)

Complications	Type of osteosynthesis Complications/total number of patients	Complications/total number of patients	Total ratio
Plate failure/pseudarthrosis	SP: 3/11 HOSO-DP:1/105	4	3.45%
Mandibular fracture after plate removal	SP: 0/11 HOSO-DP:1/105	1	0.86%
TMJ pain	SP: 1/11 HOSO-DP: 4/105	5	4,31%
Infection	SP: 0/11 HOSO-DP: 1/105	1	0.86%
Total	SP: 4/11 (36.37%) HOSO-DP: 7/105 (6.67%)	11/116	9.48%

## Discussion

The aim of the present study is to review the surgical outcomes of 116 patients that have undergone orthognathic surgery to correct mandibular prognathism, retrognathia, open bite, and asymmetries using high-oblique sagittal osteotomy (HOSO) at the Department of Oral, Maxillofacial and Facial Plastic Surgery, at Goethe University of Frankfurt, Germany. For this purpose, a retrospective analysis of the patients’ charts and their medical images was performed. This study specially focused on the development of the HOSO since its introduction to the clinic and the complications encountered during the follow-up period and those reported up until the time of this study.

The results showed that the highest number of patients operated on with the HOSO was in the age range 20–29. These results are in accordance with previous reports of patients undergoing orthognathic surgery mainly in their 20s and predominantly females [11, 12]. Additionally, the patients operated on with the HOSO in the same age range also had a higher rate of post-operative complications. In a comprehensive review involving 8390 patients operated on with the BSSO, it was also found that on average, patients in their 20s had a higher rate of post-operative complications [12]. A relationship between age and complications in orthognathic surgery has been reported in the literature; nevertheless, age-associated complications remain a controversial matter.

Several advantages have been associated with the HOSO when compared with the BSSO, for example, the preservation of the inferior alveolar nerve, a shorter intraoperative time due to the ease of this procedure, and a lower risk of unwanted fractures during the intervention [4, 6]. Furthermore, it has been previously shown that the implementation of the dedicated ramus plates reduces the intraoperative time [4]. This statement was confirmed by the present study. The results of our study showed that the patients who were operated on with only an HOSO without additional procedures had a significant reduction in OP-time and that the time was reduced over the years (*P* < 0.0015). This is of importance because a lower intraoperative time is correlated with lower blood loss and fewer complications [13, 14].

In general, the literature reports 10 ± 2% of post-operative complications associated with the BSSO [15]. The most commonly reported post-operative complications are relapse (0.2%), infection (3.4%), fixation material failure (2.5%), and neurosensory deficits (12.1%) with a permanent sensory loss of 1.8% [12]. The data gathered in this study showed, in general, a 9.4% rate of complications among patients operated on with the HOSO. However, the rate of complication reduces to 6.67% in the group of patients where only the HOSO-DP was used. TMJ pain and reduction of maximal mouth opening at 3.8% was shown to be the most common post-operative complication. It is noteworthy that no sensory alterations were reported during the long follow-up. In a similar study involving 17 patients operated on with the HOSO, at 3 months of follow-up none of the patients reported numbness or paresthesia [6]. Moreover, contrary to our results, Seeberger et al. reported that after operations on 50 patients with the HOSO, no disorders of the TMJ developed [5]. However, in the aforementioned study, all patients were operated on using the HOSO-DP. The majority of complications in our study were associated with the type of plates and the height at which the osteotomy was performed (Fig. 2c). Mainly it was the use of SP (straight plates X and Y plates) which were associated with complications and material failure. Additionally, in a previous study using a computer biomodel, the area of higher stress and dislocation after the HOSO surgery was located in the proximal segment, specifically in the coronoid process [3]. The results observed here support this assumption. In the cases found with complications associated with plate failure, the coronoid process was coronally displaced (Fig. 2a).

Once the use of the HOSO-DP was established, the rate of complications was considerably reduced. Additionally, the correct placement of the osteotomy and positioning of the plate seem to be primordial (Fig. 2). A recent biomechanical study evaluated if there was a correlation between the osteotomy angle of the HOSO with respect to the tension generated in the mandibular segments and the variation of the bone surface. Seventy-two simulations were performed under different mandibular mobilization. The results showed that there is increased stress over the mandibular segments as the angle of the osteotomy increases and that the contact between bone segments varies depending on the osteotomy angle, increasing to 44.67% from 45 to 70° and decreasing to 22.05% when the angle is reduced to 30° [16].

In many European countries, removing asymptomatic osteosynthesis plates is a routine praxis 8 to 12 months after orthognathic surgery [17]. The decision is based on the prevention of possible complications due to the exposure of plates to forces of the masticatory function [18]. In the BSSO, bone stability after plate removal has not been reported to be a problem [17, 18]. To our knowledge, no studies can be found in the literature regarding osteosynthesis plate removal associated with the HOSO. It was observed in our study that bone remodeling after a HOSO could result in a thinner mandibular ramus in the area where the bone was osteotomized. It can be inferred that after bone healing, the resulting anatomy is dependent on the quantity of advancement or setback. In the present study, one case of mandibular fracture and pseudarthrosis occurred after the removal of the osteosynthesis plates 8 month post-OP (Fig. 2d). A wide sagittal movement could result in a reduced contact area and might explain this complication. Möhlhenrich et al. calculated the bony contact area of the mandibular segments after performing an HOSO using a computer biomodel. Computer topographies of 40 patients were virtually operated on with mandibular setback and advancement. The results showed a mean area of contact with the bony surface, after 8 mm advancement for a HOSO, of 217.17 mm^2^, and after 10 mm setback of 202.64 mm^2^. Comparably with the aforementioned sagittal movements, R. Kuehle et al. reported in a clinical study a mean mandibular advancement of 6.51 mm and mean mandibular setback of 4.16 mm without observed complications [19]. Additionally, a previous study involving 8 patients operated on with the HOSO reported that complete healing of the osteotomy and excellent remodeling of the ramus was observed during plate removal [6]. A limitation of retrospective studies is the absence of determinant parameters. In the present work not all parameters of interest could be retrieved from our electronic record system, i.e., the exact distance of mandibular movement, and hence, we are unable to correlate failure of the osteosynthesis material to the extent of mandibular movement. However, in a clinical study, 33 patients were operated on using the HOSO-DP and mean advancement movements of 7.8 ± 7.1, setback of 7.7 ± 4.1, clockwise rotation of 6.3 ± 5.1 and counterclockwise rotation of 6.5 ± 7.9 were reported. In the study, no osteosynthesis fractures were seen after a follow up of 1 year [4]. Future prospective studies have to show if there is a correlation between those parameters.

**Fig. 5:**
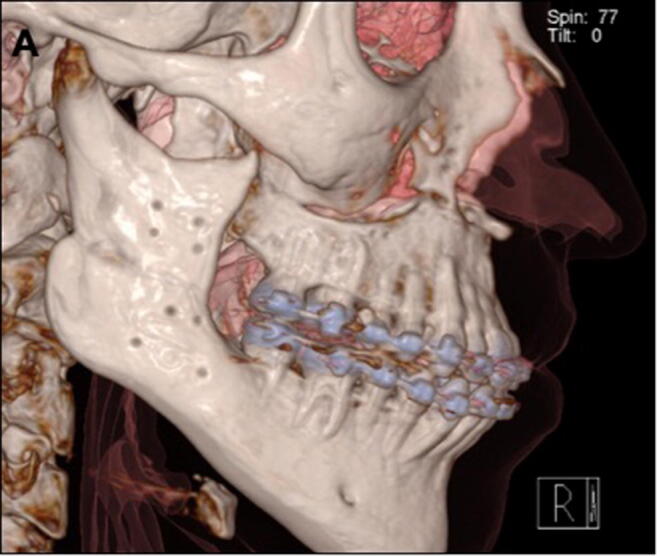
Mandibular ramus remodeling after a high-oblique split osteotomy (HOSO). Post-operative image (3D reconstruction) 8 months posterior to the removal of a dedicated ramus plate

As aforementioned, after bone healing, the borders of the ascending ramus of the mandible are remodeled (Fig. 5). Previous studies have reported that bone remodeling after a BSSO induces changes to the soft tissue [20]. To date, no such study has been performed regarding the HOSO. Despite the available literature, further studies are necessary to establish guidelines regarding the maximum sagittal movements, risk of plate removal, and the impact of bone remodeling after a HOSO on the facial profile.

## Conclusion

The original design of the high-oblique sagittal osteotomy (HOSO) was described more than 90 years ago. Nevertheless, this design of osteotomy is not considered by many surgeons as an alternative for treating patients. In fact, in the literature, little information can be found regarding the HOSO. The rate of complications in the HOSO is shown to be comparable to the rate of complications from the BSSO reported in the literature. Moreover, the use of the ramus dedicated plate appears to provide enough stability to the bone segments, making the surgery safer. However, other factors (i.e., the correct time of plate removal after bone healing, bone remodeling, and its impact on the facial profile) require further research.

## References

[1] Oh J-S, Kim S-G (2015). In vitro biomechanical evaluation of fixation methods of sagittal split ramus osteotomy in mandibular setback. J Craniomaxillofac Surg.

[2] Obwegeser H (1963). The indications for surgical correction of mandibular deformity by the sagittal splitting technique. Br J Oral Surg.

[3] Herrera-Vizcaino C, Herrera Vizcaíno M, Pelliccioni O (2016) Biomechanical simulation of different bone fixation methods with osteosynthesis material used in Schlössmann modified osteotomy. XIII Congr Int Métodos Numéricos en Ing y Ciencias XIII:113

[4] Landes C, Tran A, Ballon A, Santo G, Schübel F, Sader R (2014). Low to high oblique ramus piezoosteotomy: A pilot study. J Craniomaxillofac Surg.

[5] Seeberger R, Asi Y, Thiele OC, Hoffmann J, Stucke K, Engel M (2013). Neurosensory alterations and function of the temporomandibular joint after high oblique sagittal split osteotomy: an alternative technique in orthognathic surgery. Br J Oral Maxillofac Surg.

[6] Kaduk WMH, Podmelle F, Louis PJ (2012). Revisiting the supraforaminal horizontal oblique osteotomy of the mandible. J Oral Maxillofac Surg.

[7] Sahoo NK, Kaur P, Roy ID, Sharma R (2017) Complications of sagittal split ramus osteotomy. J. Oral Maxillofac. Pathol. 29:100–104. 10.1016/j.ajoms.2016.09.006

[8] Seeberger R, Thiele OC, Mertens C, Hoffmann J, Engel M (2013). Proximal segment positioning with high oblique sagittal split osteotomy: indications and limits of intraoperative mobile cone-beam computerized tomography. Oral Surg Oral Med Oral Pathol Oral Radiol.

[9] Möhlhenrich SC, Kamal M, Peters F, Fritz U, Hölzle F, Modabber A (2016). Bony contact area and displacement of the temporomandibular joint after high-oblique and bilateral sagittal split osteotomy: A computer-simulated comparison. Br J Oral Maxillofac Surg.

[10] Agha RA, Borrelli MR, Farwana R, Koshy K, Fowler AJ, Orgill DP, Zhu H, Alsawadi A, Noureldin A, Rao A, Enam A, Thoma A, Bashashati M, Vasudevan B, Beamish A, Challacombe B, de Wilde RL, Machado-Aranda D, Laskin D, Muzumdar D, D'cruz A, Manning T, Healy D, Pagano D, Goel P, Ranganathan P, Pai PS, Raja S, Ather MH, kadioäžlu H, Nixon I, Mukherjee I, Gómez Rivas J, Raveendran K, Derbyshire L, Valmasoni M, Chalkoo M, Raison N, Muensterer O, Bradley P, Roberto C, Afifi R, Rosin D, Klappenbach R, Wynn R, Giordano S, Basu S, Surani S, Suman P, Thorat M, Kasi V (2018). The PROCESS 2018 statement: updating consensus Preferred Reporting Of CasE Series in Surgery (PROCESS) guidelines. Int J Surg.

[11] Kim S-G, Park S-S (2007). Incidence of complications and problems related to orthognathic surgery. J Oral Maxillofac Surg.

[12] Sousa CS, Turrini RNT (2012). Complications in orthognathic surgery: a comprehensive review. J Oral Maxillofac Surg Med Pathol.

[13] Andersen K, Thastum M, Nørholt SE, Blomlöf J (2016). Relative blood loss and operative time can predict length of stay following orthognathic surgery. Int J Oral Maxillofac Surg.

[14] Tsai CY, Chang YJ, Wu TJ, Lai JP, Chen TY, Lin SS (2019). Blood loss and operative time associated with orthognathic surgery utilizing a novel navigation system in cleft lip and palate patients. J Formos Med Assoc.

[15] Olate S, Sigua E, Asprino L, De Moraes M (2018). Complications in orthognathic surgery. J Craniofac Surg.

[16] Herrera-Vizcaino C, Baselga Lahoz M, Pelliccioni Monrroy O, et al (2020) Stress distribution is susceptible to the angle of the osteotomy in the high oblique sagittal osteotomy (HOSO): biomechanical evaluation using finite element analyses. Comput Methods Biomech Biomed Engin 0:1–9. 10.1080/10255842.2020.1810242 10.1080/10255842.2020.1810242 32845167

[17] Sukegawa S, Kanno T, Manabe Y, Matsumoto K, Sukegawa-Takahashi Y, Masui M, Furuki Y (2018). Is the removal of osteosynthesis plates after orthognathic surgery necessary? Retrospective long-term follow-up study. Int J Oral Maxillofac Surg.

[18] Rauso R, Tartaro G, Stea S, Tozzi U, Biondi P (2011). Plates removal in orthognathic surgery and facial fractures: When and why. J Craniofac Surg.

[19] Kuehle R, Berger M, Saure D, Hoffmann J, Seeberger R (2016). High oblique sagittal split osteotomy of the mandible: assessment of the positions of the mandibular condyles after orthognathic surgery based on cone-beam tomography. Br J Oral Maxillofac Surg.

[20] Meulstee J, Liebregts J, Xi T, Vos F, de Koning M, Bergé S, Maal T (2015). A new 3D approach to evaluate facial profile changes following BSSO. J Craniomaxillofac Surg.

